# The impact of psychosocial factors on the risk of erectile dysfunction and inhibition of sexual desire in a sample of the Brazilian population

**DOI:** 10.1590/S1516-31802005000100003

**Published:** 2005-01-02

**Authors:** Carmita Helena Najjar Abdo, Waldemar Mendes de Oliveira Júnior, Edson Moreira Júnior, João Afif Abdo, João Antônio Saraiva Fittipaldi

**Keywords:** Impotence, Depression, Education, Unemployment, Impotência, Depressão, Educação, Desemprego

## Abstract

**CONTEXT:**

Sexual dysfunctions can have origins in physical, psychological and psychosocial factors.

**OBJECTIVE:**

To describe the frequency of erectile dysfunction (ED) and female inhibition of sexual desire (ISD) in a Brazilian sample, and to estimate the risks of these dysfunctions.

**TYPE OF STUDY:**

Non-random survey.

**SETTING:**

Ten Brazilian cities.

**METHODS:**

2,835 subjects (53% women) aged over 18 years answered a questionnaire about their general health and sex life. The chi-squared test and multivariate logistic regression were used. Values of p ≤ 0.05 were considered significant.

**RESULTS:**

The women’s average age was 36.6 years (± 13.3) and the men’s was 39.5 (± 13.3). 14.7% of men presented moderate/complete ED and 34.6% of women presented ISD. Depression was mentioned by 16.8% of men and 29.7% of women. The chances of having ED and ISD were higher for subjects who had had lower school attainment. Lack of a job and depression gave rise to 1.5 times (95% CI: 1.0 – 2.3) and 1.9 times (95% CI: 1.2 – 3.0) greater chances of ED respectively. Compared with men aged up to 25 years, those aged 41-60 had 1.9 times (95% CI: 1.0 – 3.4) and those aged 61 and over had 5.4 times (95% CI: 2.3 – 12.6) greater risk of ED. For women, lack of a job gave rise to 1.5 times (95% CI: 1.1 – 1.9) greater chance of ISD; depression was not associated with higher risk. Compared with women aged up to 25 years, those aged 41-60 and 61 or over had, respectively, 2.9 times (95% CI: 2.0 – 4.1) and 7.5 times (95% CI: 3.0 –18.6) greater risk of ISD.

**DISCUSSION:**

Increasing unemployment has affected the whole population, but especially those with lower levels of school attainment. Such levels are positively linked with presence of sexual dysfunctions.

**CONCLUSION:**

Lack of jobs, age and low school attainment are risks for the sexual dysfunctions studied. Depression increased the risk of ED but not female ISD.

## RESUMO

**CONTEXTO:**

As disfunções sexuais originam-se de fatores físicos, psicológicos e psicossociais.

**OBJETIVO:**

Apresentar freqüências de disfunção erétil e inibição do desejo sexual feminino em amostra brasileira e estimar riscos destas disfunções.

**TIPO DO ESTUDO:**

Transversal.

**LOCAL:**

10 cidades brasileiras.

**MÉTODOS:**

2.835 indivíduos (53% mulheres), maiores de 18 anos, responderam a um questionário sobre saúde geral e vida sexual. Foram utilizados testes de qui-quadrado e regressão logística multivariada. São significantes valores de p ≤ 0.05.

**RESULTADOS:**

A idade média foi 36,6 anos (DP = 13,3) para mulheres e 39,5 anos (DP = 13,3) para homens. 14,7% dos homens apresentavam disfunção erétil moderada/completa (DE) e 34,6% das mulheres, inibição do desejo sexual (IDS). Depressão foi referida por 16,8% dos homens e 29,7% das mulheres. A chance para DE e IDS foi maior para indivíduos com menos anos de escolaridade. Falta de emprego e depressão aumentaram 1,5 vezes (95% CI; 1,0 – 2,3) e 1,9 vezes (95% CI; 1,2 – 3,0) as chances para DE, respectivamente. Comparados aos homens com até 25 anos de idade, aqueles com 41 a 60 anos tiveram risco 1,9 vezes maior para DE (95% CI; 1,0 – 3,4) e 5,4 vezes (95% CI; 2,3 – 12,6) para aqueles com 61 anos ou mais. Para mulheres, a falta de emprego aumentou 1,5 vezes a chance para IDS (95% CI; 1,1 – 1,9); a depressão não aumentou o risco. Comparadas às mulheres com até 25 anos, aquelas com 41 a 60 anos e com 61 anos ou mais têm risco 2,9 vezes maior para IDS (95% CI; 2,0 – 4,1) e 7,5 vezes maior (95% CI; 3,0 – 18,6), respectivamente.

**DISCUSSÃO:**

O desemprego, como tendência mundial crescente, tem afetado todos os níveis da população, especialmente os com baixo nível de escolaridade. Baixos níveis de escolaridade estão correlacionados positivamente à presença de disfunções sexuais.

**CONCLUSÕES:**

Ausência de emprego, idade e baixa escolaridade representam risco para as disfunções sexuais pesquisadas. Depressão aumentou o risco para disfunção erétil, mas não para inibição do desejo sexual feminino.

## INTRODUCTION

Sexual dysfunctions are represented by disturbances related to sexual desire and psycho-physiological changes in the cycle of sexual response. Such dysfunctions cause great suffering, as well as interpersonal difficulties.^1^

About 30 million men in the United States present some erectile dysfunction that causes suffering and interpersonal difficulties.^1-4^ According to the Massachusetts Male Aging Study (MMAS),^5^ 52% of men between 40 and 70 years old present some type of erectile dysfunction. In a recent cross-national study published in 2003, 34% of Japanese men, 22% of Malaysians, 17% of Italians and 15% of Brazilians were found to present some moderate to severe erectile dysfunction.^6^

The interest in sexual dysfunctions that has arisen both among health professionals and among the general public is partly due to increasing knowledge of the neurovascular mechanisms of the sexual response in men and women.^7-9^ Several types of drugs have been developed for the treatment of erectile dysfunction, while other agents have been suggested for the treatment of inhibition of sexual desire.^10-14^ However, few studies on the origin of these disturbances have investigated psychosocial factors such as unemployment. The increasing risk of erectile dysfunction has been correlated with diabetes, coronary heart disease and smoking.^5,6,15-17^ A risk of erectile dysfunction has also been found among depressed and unemployed men.^6,15,18-24^

Inhibition of sexual desire, defined as the absence of or a severe decrease in fantasies and desires related to any sexual activity, is the most frequent sexual dysfunction among women.^17,25-27^ The prevalence of female inhibition of sexual desire ranges from 5% to 46%.^25^ Nathan (1986) found rates of 1% to 35% among women with complaints of inhibition of sexual desire in a literature review.^28^ Depression and age are the most common factors in the development of this dysfunction in women, as shown in several studies.^29-31^

The present study shows the frequencies of depression, erectile dysfunction and inhibition of sexual desire in a sample of the Brazilian population, with the aim of evaluating the risk of erectile difficulties and inhibition of sexual desire in relation to depression, unemployment, aging and education level.

## METHODS

The Brazilian Study of Sexual Behavior (BSSB) was conducted between February and April 2000, and was performed using a non-random sample of 2,835 subjects (53% were women), from ten cities in seven Brazilian states.^32^ All subjects were aged 18 years or over. The sample was comparable to the general population of Brazil in terms of ethnicity and religion, but had a larger proportion of subjects with higher education levels.^33^The subjects were weekend visitors to beaches, parks and shopping centers, and they were invited to answer an anonymous self-administered questionnaire.

The original questionnaire was submitted to a pilot test, in which 30 subjects of both sexes answered it. Following adjustments in terms of consistency and reliability, the final questionnaire included questions on demographic data (e.g. age, employment and education level), general health (self-reporting of medical conditions), lifestyle and sexual behavior. The questionnaire has already been published elsewhere.^32^

With regard to the erectile dysfunction assessment, the subjects were requested to choose one category that best described them: always, usually, sometimes, or never able to achieve and maintain an erection that was satisfactory for sexual performance. The answers were then used to classify the respondents into one of the following categories: none, minimal, moderate, or complete erectile dysfunction, respectively.

For the assessment of inhibition of sexual desire and depression, subjects were asked to mark with an "X", beside the respective question, if there had been some inhibition or absence of sexual desire or depression during their lives.

The chi-squared test and multivariate logistic regression were used for statistical analysis. Values of p ≤ 0.05 were considered statistically significant.

Not all the men or women answered all the items of the questionnaire. Consequently, in presenting the variable studied, the number of subjects who answered this question has been specified. There were no differences between subjects who answered these questions and those who did not.

For the multivariate logistic models, there were 1,257 women and 1,057 men. Only the variables that were part of the main objective of the present study (age, education level, unemployment and depression) have been included. Men and women who did not answer at least one of the questions on age, education level, depression or employment have been excluded from this study. No statistically significant difference was presented between those who were excluded and those included, in relation to the variables studied.

## RESULTS

The average age was 36.6 years (standard deviation, SD = 13.3) for women and 39.5 years (SD = 13.3) for men.

41% of the women and 22.5% of the men who answered the question about working condition were unemployed. Depression was mentioned by 29.7% of the women and 16.8% of the men who answered this questions.

The total frequency of feminine inhibition of sexual desire was 34.6% of respondents. For men, 14.7% had moderate to severe erectile dysfunction.

We observed that unemployed women and men presented higher frequencies of depression, as well as higher frequencies of inhibition of sexual desire and erectile dysfunction, respectively (Table 1).

**Table 1:** Proportion (%) of depression and sexual dysfunctions (inhibition of sexual desire in women or moderate/complete erectile dysfunction in men) according to age and working status and education attainment by gender

	Women		Men	
	Total	ISD n (%)	Total	ED n (%)
***Age (years)***				
Up to 25	384	90 (23.4)	190	19 (10.0)
26 to 40	561	145 (25.8)	493	47 (9.5)
41 to 60	455	214 (47.0)	395	71 (18.0)
> 60	74	54 (73.0)	83	32 (38.6)
p for trend		< 0.01		< 0.01
	**Women**		**Men**	
	**Dep n (%)**	**ISD n (%)**	**Dep n (%)**	**ED n (%)**
***Working status***				
Unemployed	175/526 (33.3%)	189/526 (35.9%)	55/263 (20.9%)	43/240 (17.9%)
Employed	202/757 (26.7%)	205/757 (27.1%)	146/905 (16.1%)	93/822 (11.3%)
p for trend	0.011	0.001	0.07	0.007
	**Women**		**Men**	
	n (%)		n (%)	
***Educational attainment (years)***				
Less than eight	118 (8.0)		93 (7.3)	
Eight	156 (10.6)		138 (10.8)	
Eleven	604 (41.1)		537 (41.9)	
More than eleven	591 (40.2)		514 (40.1)	
p for gender	0.884			

*ISD = inhibition of sexual desire; ED = erectile dysfunction; Dep = depression.*

Both erectile dysfunction and inhibition of sexual desire are directly related to aging. As Table 1 shows, there is a tendency towards higher frequencies of these dysfunctions among older subjects.

The results from the multivariate logistic regression model showed that, for women, being unemployed, getting older and having less than 11 years of school education increased the chance of inhibition of sexual desire (Table 2). Depression did not increase the chance of inhibition of sexual desire and was not included in the final model.

**Table 2:** Odds ratios and 95% confidence interval (CI) for female inhibition of sexual desire (n = 1,257) and erectile dysfunction (n = 1,057), according to age, social-demographic characteristics and depression*

	Female inhibition of sexual desire	Erectile dysfunction
Variable	Odds ratio (95% CI)	Odds ratio (95% CI)
**Age (years)**		
Up to 25	1.0 (reference)	1.0 (reference)
26-40	1.1 (0.8 – 1.6)	0.9 (0.5 – 1.7)
41-60	2.9 (2.0 – 4.1)	1.9 (1.0 – 3.4)
> 60	7.5 (3.0 – 18.6)	5.4 (2.3 – 12.6)
**Educational attainment (years)**		
More than eleven	1.0 (reference)	1.0 (reference)
Eleven years	1.3 (1.0 – 1.8)	1.8 (1.1 – 2.8)
Eight years	2.4 (1.5 – 3.7)	2.8 (1.5 – 5.3)
Less than eight	1.8 (1.1 – 3.0)	4.4 (2.2 – 8.7)
**Working status**		
Employed	1.0 (reference)	1.0 (reference)
Unemployed	1.5 (1.1 – 1.9)	1.5 (1.0 – 2.3)
**Depression**		
No		1.0 (reference) 1.9 (1.2 – 3.0)
Yes		

* *Data obtained from the Brazilian Study of Sexual Behavior. Results derived from multivariate logistic regression.*

Unemployment, aging, less than 11 years of school education and depression increased the chance of moderate to severe erectile dysfunction among men (Table 2).

## DISCUSSION

The frequencies of erectile dysfunction and inhibition of sexual desire in our sample were high.

In the medical literature, different prevalences of sexual dysfunctions are found. This variation is due partly to the type of sample studied (community *versus* sexuality clinics), the type of trial performed (self-administered questionnaires or interview), and the clinical definition used for each dysfunction.^25^

The present study does not represent a sample of all socioeconomic-cultural classes of the Brazilian population, but it can give us an idea of how prevalent sexual dysfunctions are. It can serve as a starting-point for future surveys with a wider-ranging sample of people and also for pointing out possible associations between sexual dysfunctions and depression and other sociodemographic factors.

The same tendency towards increasing erectile dysfunction and inhibition of sexual desire with age that was noticed in previous studies was found in the present sample.^3,6,15,17,24^

Several studies have indicated a relationship between depressive symptoms and erectile dysfunction. These conditions are commonly associated, mutually maintaining or enhancing each other.^34-37^

The trend towards increasing unemployment around the world^18^ has affected all levels of the population, especially in Brazil. It has particularly affected those with lower levels of school attainment. On the other hand, lower levels of education are also positively linked with the presence of sexual dysfunctions in the present sample, in the same way as found by Laumann, Paik and Rosen (1999).^17^ In our sample, these two factors worked independently to increase the chances of having the sexual dysfunctions studied. Therefore, the higher risk of sexual dysfunction among unemployed subjects cannot be explained by the low level of school attainment alone.

## CONCLUSIONS

Age, unemployment and low levels of school attainment increased the risk of inhibition of sexual desire among women and erectile dysfunction among men independently of each other, in the present sample.

Depression increased the risk of erectile dysfunction, but not female inhibition of sexual desire, which suggests a need for new investigation and analysis.

The results from this study show that, for correct diagnosis and successful treatment of these dysfunctions, emotional factors (depression) and socioeconomic-cultural factors must be assessed alongside physical factors.

Older patients’ own characteristics should also be taken into consideration, since the older the subjects are, the more prevalent these dysfunctions are.

This study has confirmed the strong association between sexual dysfunctions, depression and poor socioeconomic conditions such as unemployment and low educational level.
